# Efficacy of a Transdiagnostic Self-Help Internet Intervention for Reducing Depression, Anxiety, and Suicidal Ideation in Adults: Randomized Controlled Trial

**DOI:** 10.2196/22698

**Published:** 2021-01-22

**Authors:** Philip J Batterham, Alison L Calear, Louise Farrer, Amelia Gulliver, Ella Kurz

**Affiliations:** 1 Centre for Mental Health Research Research School of Population Health Australian National University Canberra ACT Australia

**Keywords:** depression, anxiety, randomized controlled trial, internet, implementation science

## Abstract

**Background:**

Low-intensity self-guided mental health interventions that are delivered on the web may meet the needs and preferences of adults with mild to moderate symptoms. However, few clinical trials have examined the effectiveness of self-guided transdiagnostic interventions within a naturalistic setting.

**Objective:**

This randomized controlled trial (RCT) tests the effectiveness of the video-based transdiagnostic intervention *FitMindKit* in reducing depression symptoms (primary outcome), anxiety symptoms, disability, and suicidal ideation, relative to an attention-matched control condition called *HealthWatch*.

**Methods:**

The RCT was conducted with adults living in the Australian Capital Territory, Australia. Participants (n=1986) were recruited through the web using social media advertisements, screened for psychological distress, and then randomized to receive one of two 4-week programs: *FitMindKit* (12-module psychotherapy intervention) or *HealthWatch* (12-module program providing general health information). Participants were assessed at baseline and at 4 weeks postbaseline. To maintain the ecological validity of the trial, participants completed brief assessments and interventions without direct researcher contact or incentives.

**Results:**

Mixed model repeated-measures analyses of variance demonstrated that *FitMindKit* significantly improved depression symptoms (*F*_1,701.7_=3.97; *P*=.047), along with panic symptoms (*F*_1,706.5_=5.59; *P*=.02) and social anxiety symptoms (*F*_1,680.0_=12.37; *P*<.001), relative to the attention control condition. There were no significant effects on other outcomes.

**Conclusions:**

Self-guided transdiagnostic interventions can be beneficial when delivered directly to end users through the internet. Despite low adherence and small effect sizes, the availability of such interventions is likely to fill a critical gap in the accessibility of mental health services for the community.

**Trial Registration:**

Australian New Zealand Clinical Trials Registry ACTRN12618001688279; http://www.anzctr.org.au/Trial/Registration/TrialReview.aspx?id=376113.

**International Registered Report Identifier (IRRID):**

RR2-10.1016/j.conctc.2019.100341

## Introduction

Depression and anxiety are common mental disorders that account for significant global disease and disability burden [1]. Despite significant personal, interpersonal, social, educational, and vocational impacts of these disorders, only one in 3 people with a mental health problem seeks help from a health professional [2-4]. Those who engage in professional treatment typically delay seeking help for years or decades [5]. Cost of treatment, stigma, lack of knowledge, and low perceived need for treatment are commonly cited as barriers to seeking mental health help [6-8]. Internet-based, self-guided mental health interventions are able to mitigate some of these barriers and have been shown to be effective, efficient, and cost-effective in the prevention and treatment of many common mental disorders [9-11]. The high rate of comorbidity between depression, anxiety, and other mental health problems represents another significant challenge for help seeking and treatment, as it is often related to an increase in the severity and chronicity of symptoms [12,13]. One solution to the challenge of treating comorbid mental health problems is the use of transdiagnostic treatment approaches. Transdiagnostic treatments are typically based on the principles of cognitive behavioral therapy (CBT), which is an efficacious treatment approach for multiple mood and anxiety disorders [14,15]. Transdiagnostic treatments provide therapeutic content capable of targeting the common cognitive and behavioral processes that underlie the development and maintenance of multiple mental health disorders (eg, negative thinking patterns, avoidance, hyperarousal) and address symptoms that are common to multiple disorders (eg, sleep disturbance) [16,17].

Internet-based transdiagnostic mental health interventions have the potential to overcome barriers to mental health help seeking and treatment, and there is extensive evidence for their effectiveness [17-19]. There is evidence that such interventions may be more effective and efficient than interventions focusing on a single disorder [15]. However, the potential of web-based interventions to address comorbidity has not been fully realized. Trials of transdiagnostic internet interventions have largely been delivered within clinical settings [17] and, with very few exceptions [20], have focused on clinician-guided interventions. The provision of clinical support in an intervention requires increased resources, thereby reducing the potential for scalability. Furthermore, delivering interventions within a clinical setting neglects the majority of the population who do not engage in clinical care for mental health problems [2]. There is meta-analytic evidence that individuals with mild to moderate symptoms of depression or anxiety may benefit from self-guided interventions [21,22], which can be delivered without the need to connect with a health professional. Furthermore, delivery of self-guided internet interventions through web-based marketing may become an important component for prevention and early intervention, as it bypasses traditional treatment settings. Distal delivery of interventions may also become more critical in the face of public health crises and other system shocks, such as pandemics [23], and natural disasters (eg, bushfires, floods), when the delivery of traditional health services is hampered because of access restrictions.

As the abovementioned studies highlight, there is a need for randomized controlled trials (RCTs) comparing web-based interventions that address comorbidity with a robust attention control condition. As outlined in the protocol paper for this RCT [24], our research group has developed the *FitMindKit* program, a self-guided transdiagnostic web-based program that delivers CBT and other techniques through a series of brief videos and self-directed exercises. The program focuses on reducing both depression and anxiety symptoms using CBT-based content such as cognitive restructuring, problem-solving skills, and relaxation strategies. *FitMindKit* has been tested previously in an RCT comparing tailored and static versions of the program [25]. In that underpowered trial, there were no significant differences in effectiveness or adherence for a tailored version of the program, compared with a static version of the program. Consequently, this RCT tested a simplified static version of the program against an attention-matched control condition. This study was nested within a broader implementation study examining the uptake and reach of *FitMindKit* delivered directly to users through web-based advertising, in comparison with 2 community settings: primary care practices and pharmacies. The web-based arm of the implementation study is the focus of this paper, which used an RCT to test the effectiveness of the intervention in reducing depression symptoms.

We used a naturalistic RCT design for this study, where participants had no contact with researchers or clinicians and completed the intervention and brief assessments with only automated support (reminder emails). Although such an approach may limit adherence and increase attrition, leading to more modest effects [25-27], the methodology best reflects the delivery of self-help programs in the community [28]. Thus, the objective of this trial was to test the effectiveness of *FitMindKit* in reducing symptoms of depression and anxiety, suicidal ideation, and disability, when delivered in a real-world setting.

## Methods

### Trial Design

A two-arm parallel RCT was conducted comparing an active intervention (*FitMindKit)* with an attention control condition (*HealthWatch)*. The trial protocol is provided in Batterham et al [24].

### Ethics Approval

This trial was approved by the Australian National University Human Research Ethics Committee (ANU HREC protocol number 2017/911).

### Participants and Procedure

Participants were recruited through the web between October 2018 and August 2019 via paid social media advertisements on Facebook and Instagram. Advertisements targeted adults aged 18 years and older living in the Australian Capital Territory to match the delivery catchment for the overarching implementation trial. On clicking on the Facebook advertisement, participants were directed to a webpage containing information about the study and questions to obtain their consent to participate. At this point, participants were assessed for eligibility as described in the following section, *Eligibility Criteria*. Following eligibility screening, a baseline assessment was completed, followed by randomization and treatment allocation, and a postintervention assessment following the 4-week intervention period.

### Eligibility Criteria

To be eligible for the trial, participants were required to be aged 18 years and older and had moderate psychological distress as measured by the Distress Questionnaire-5 (DQ5) [29]. The DQ5 is a measure of psychological distress and consists of 5 items that assess the symptoms of common mental disorders. Participants were asked to endorse the frequency of each item over the last 30 days on a 5-point scale, ranging from never to always, with scores ranging from 5 to 25. In this study, the categories of DQ5 scores were no or low psychological distress (score of 5-7), moderate psychological distress/risk of a mental disorder (score of 8-17), and high-risk/probable clinical symptoms of a mental disorder (score of 18-25). These cut points were selected based on percentiles from existing population-based data [29]. Participants aged 18 years and older and who scored in the moderate risk category (score of 8-17) on the DQ5 were eligible for the trial, as a web-based self-guided mental health program is likely to be the most suitable for this group. Those in the low-risk category were provided with feedback and resources to access if their symptoms changed. Those in the high-risk category were strongly encouraged to seek help from a health professional and provided contact details for face-to-face, telephone-based, and web-based mental health resources and services.

Eligible participants were invited to access the web-based trial portal containing trial assessments and intervention and control programs. Participants were required to create an account in the portal using an email address and password and then complete a brief baseline questionnaire. Following the completion of baseline measures, participants were automatically randomized, without involvement by trial staff, to receive the active intervention (*FitMindKit*) or the attention control program (*HealthWatch*). Randomization was in a 1:1 allocation ratio by a computer-generated sequence, with a block size of 6 stratified by age, sex, and DQ5 severity score, and the trial was double blinded. Following randomization, participants were provided with access to their assigned program and instructed to complete the program modules at their own pace over a 4-week period. They were also sent an email containing details of their allocated condition and a link to the portal. During the intervention period, participants received an automated weekly email reminding them to engage with their allocated program. Following the 4-week period, participants were sent an email inviting them to complete the postintervention survey. They received 2 reminder emails if they had not completed the survey after 1 and 2 weeks.

### Interventions

#### Active Transdiagnostic Self-Guided Intervention: FitMindKit

**Figure 1:**
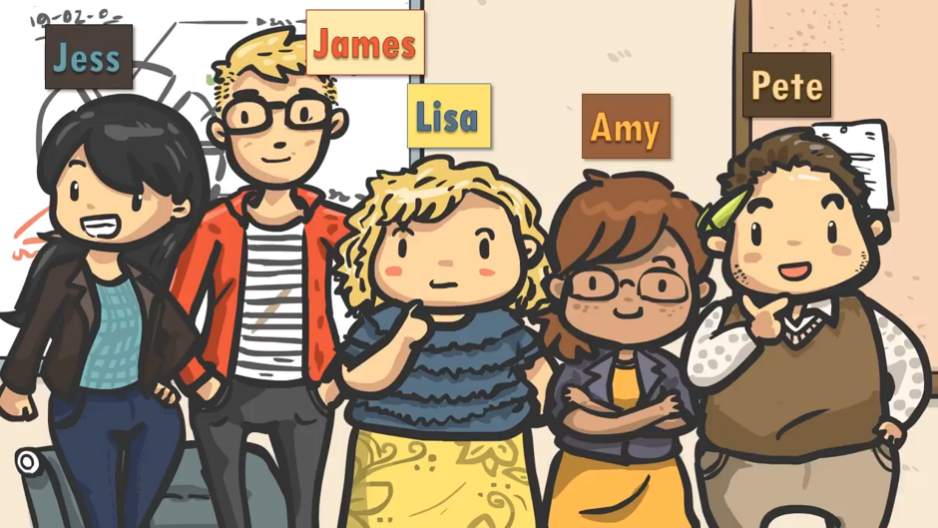
Screenshot of FitMindKit characters.

Each *FitMindKit* module consists of a 2- to 6-minute video in which a series of fictional animated characters introduce concepts of and share their personal experience of one or more mental health problems. Figure 1 depicts *FitMindKit* characters. A relevant *homework* activity designed to facilitate practice of the therapeutic technique presented accompanies each video. Participants allocated to *FitMindKit* condition were able to access all 12 modules over the 4-week trial period. In total, 8 of the modules contained therapeutic techniques based on CBT principles (psychoeducation, getting help and support, cognitive reframing, problem solving, mindfulness, managing relationships, exercise and diet, and sleep hygiene), 2 modules targeted mood (behavioral activation and reducing rumination), 1 module targeted anxiety (exposure), and 1 module targeted suicidality (distress tolerance). Participants were directed to complete the module on psychoeducation first and were then free to choose when and in which order they completed the remaining modules over the 4-week period.

#### Attention Control Condition: HealthWatch

Participants in the attention control condition received a web-based health program called *HealthWatch,* a 12-module program pertaining to general health rather than mental health, which is not associated with therapeutic reductions in depression. Modules are text based and contain information on respiratory viruses, heart health, microbes, bone health, food hygiene, sun exposure, dietary supplements, kidney health, household burns, allergens, pancreas health, and posture. Participants randomized to the *HealthWatch* program were able to access the 12 modules over a 4-week period and complete them in any order.

### Outcome Measures

The Patient Health Questionnaire-9 (PHQ-9) was the primary outcome measure, assessing the frequency of symptoms of major depression [30]. This scale consists of 9 items rated on a 4-point scale, ranging from *not at all* to *nearly every day*, and item scores are summed to produce an overall severity score ranging from 0 to 27, with higher scores indicating higher symptom severity. The PHQ-9 has sound sensitivity and specificity for detecting major depression in both clinical and general population samples and has been shown to detect change over time [31]. Internal consistency was adequate in the current sample at baseline (α=.78).

The Generalized Anxiety Disorder Scale-7 (GAD-7) was used to assess symptoms of generalized anxiety disorder (GAD) according to *Diagnostic and Statistical Manual of Mental Disorders* (DSM)-IV and DSM-5 diagnostic criteria [32]. The 7 items from the scale are rated on the same 4-point scale as the PHQ-9 and summed scores on the GAD-7 range from 0 to 21, with higher scores indicating greater symptom severity. Studies have demonstrated that the GAD-7 has good psychometric properties in general population samples [31,33]. Internal consistency was good in the current sample (α=.82).

Symptoms of panic and social anxiety were measured using the 4-item Panic Disorder Screener and the 4-item Social Anxiety Disorder Screener, respectively [34]. These scales have been developed and validated using Australian community–based samples, and they assess the frequency of panic symptoms (eg, “I had a sudden unexpected period of intense fear, anxiety or discomfort”) and social anxiety symptoms (eg, “I felt nervous during social situations”) in the past 30 days. Items are rated on a 5-point scale, ranging from *never* to *always.* Both screeners have demonstrated good convergent and divergent validity with diagnostic measures [34] and had good internal consistency in this sample (panic: α=.83; social anxiety: α=.91).

Suicidal ideation was measured using the Suicidal Ideation Attributes Scale (SIDAS) [35]. The SIDAS contains 5 items assessing the frequency of suicidal ideation, controllability of suicidal thoughts (reverse coded), closeness to suicide attempts, distress associated with suicidal thoughts, and functional impact of suicidal thoughts. Items are rated on a 10-point scale, and total scores range from 0 to 50, with scores greater than 21 indicating a high risk of suicidal behavior. The SIDAS has demonstrated high internal consistency and good convergent validity [35], with adequate internal consistency in this study sample at baseline (α=.76).

The extent of disability and disruption felt by participants because of mental health problems was measured with 2 items [36]: “How many days out of the past 30 were you totally unable to work or carry out your normal activities due to mental health problems?” (range 0-30) and “How many days out of the last 30 were you able to work or carry out your normal activities but had to cut back on what you did or did not get as much done as usual due to mental health problems?” (range 0-30). These items have been used previously in a large community-based epidemiological study of mental disorders in the United States [36].

Satisfaction was assessed using a 7-item scale assessing how much the participant (1) enjoyed the program, (2) found it helpful, (3) understood the content, (4) found it interesting, (5) would use it in the future, (6) would recommend it to others, and (7) “learnt” new skills from the program. Each item is rated on a 10-point scale, ranging from *completely disagree* to *completely agree*. The satisfaction scale had acceptable internal consistency in this study sample at baseline (α=.76).

### Sample Size

The target sample size was 750, based on detecting a moderate effect size of Cohen *d* of 0.4 at posttest with 90% power, assuming 30% attrition from the trial. It was evident early in the trial that the rates of attrition were much higher than anticipated; however, demand for participation in the trial was also greater than anticipated. Consequently, we exceeded the original target to ensure that the final sample was greater than the 525 required at posttest, allowing us to detect more modest effects.

### Statistical Methods

The effectiveness of the *FitMindKit* intervention was assessed using mixed model repeated-measures (MMRM) analyses of variance (ANOVA) [37] to compare mean scores on the outcome measures between the intervention and control groups. The critical test of effectiveness is the interaction between condition (intervention vs control) and time (posttest vs baseline). MMRM provides an intention-to-treat analysis, with unbiased estimates that account for all available data from participants who were enrolled in the trial [37]. An unstructured variance-covariance matrix was assumed, and df were estimated with Satterthwaite correction. The primary outcome was depression symptoms (PHQ-9) at the posttest end point. Alpha was set at *P*<.05 for MMRM analyses. Evidence for moderation of outcomes by gender, age group, educational attainment, and module completion was tested using MMRM analyses with three-way interactions of moderator×time×condition. Alpha was set at *P*<.01 for moderation analyses to account for multiple comparisons. Finally, satisfaction was compared across the 2 conditions on the basis of a two-tailed *t* test.

## Results

**Figure 2:**
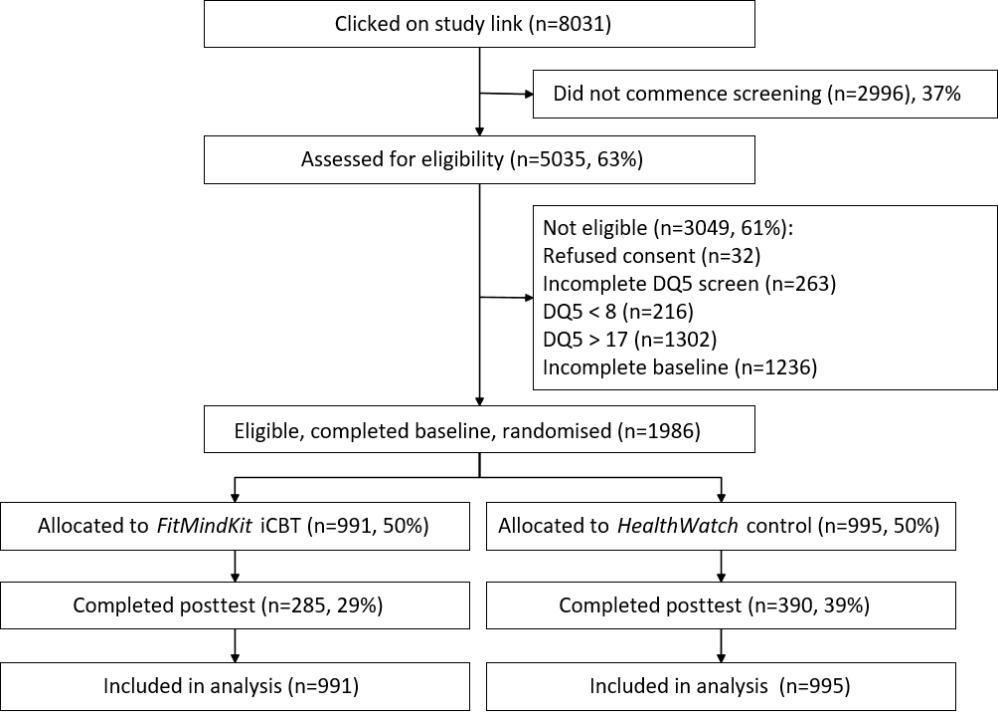
CONSORT (Consolidated Standards of Reporting Trials) flow diagram. DQ5: Distress Questionnaire-5; iCBT: internet-based cognitive behavioral therapy.

A total of 1986 adults were recruited and randomized into the study between October 2018 and September 2019. Table 1 presents the characteristics of the sample by trial condition. The sample predominantly comprised females aged between 26 and 55 years, who were employed full-time, and from an English-speaking background. Figure 2 depicts the CONSORT (Consolidated Standards of Reporting Trials) flow diagram for the trial. Considerable attrition from the trial was observed, with only 34.19% (679/1986) of participants across both conditions completing the posttest assessment. Although older participants were more likely to complete the posttest (χ*^2^*_5_=32.4; *P*<.001), there were no differences in attrition by gender (χ*^2^*_3_=0.5; *P*=.93) or education (χ^2^_9_=12.9; *P*=.17). Similarly, there were no differences in attrition by any of the baseline symptom measures (*P*>.05), except for a difference in suicidal ideation severity (completers: mean 7.09, SD 9.04; dropouts: mean 6.12, SD 8.54; t_1826_=2.24; *P*=.03). The proportion of participants who completed the posttest assessment was significantly higher in the control condition (391/995, 39.5%) than in the intervention condition (288/991, 28.9%; *P*<.001). Intervention adherence was also low—participants accessed a mean of 2.2 (SD 3.4) of the 12 *FitMindKit* modules, with 13.4% (133/991) completing 6 or more modules. In contrast, participants in the HealthWatch control condition completed a mean of 6.2 (SD 5.1) modules. There were no differences in demographic or outcome variables between conditions at baseline.

**Table 1:** Sample characteristics based on condition.

Characteristic		FitMindKit (intervention; n=991), n (%)	HealthWatch (attention control; n=995), n (%)	Chi-square (*df*)	*P* value
**Gender**				0.4 (3)	.95
	Male	141 (14.2)	136 (13.7)		
	Female	841 (84.9)	850 (85.4)		
	Other	3 (0.3)	4 (0.4)		
	Prefer not to answer	6 (0.6)	5 (0.5)		
**Age group (years)**				8.0 (5)	.16
	18-25	134 (13.5)	134 (13.5)		
	26-35	279 (28.2)	232 (23.3)		
	36-45	214 (21.6)	219 (22.0)		
	46-55	204 (20.6)	223 (22.4)		
	56-65	121 (12.2)	134 (13.5)		
	66+	39 (3.9)	53 (5.3)		
**Education**				1.0 (4)	.92
	High school or less	191 (19.3)	184 (18.5)		
	Certificate, diploma, or associate degree	242 (24.4)	258 (25.9)		
	Bachelor’s degree	260 (26.2)	265 (26.6)		
	Higher degree	294 (29.7)	285 (28.6)		
	No answer	4 (0.4)	3 (0.3)		
**Employment status**				2.6 (4)	.63
	Full-time	519 (52.4)	494 (49.6)		
	Part-time/casual	255 (25.7)	257 (25.8)		
	Unemployed	69 (7.0)	82 (8.2)		
	Not working	133 (13.4)	143 (14.4)		
	Prefer not to answer	15 (1.5)	19 (1.9)		
**Language spoken at home**				0.7 (1)	.42
	English only	902 (91.0)	895 (89.9)		
	Other	89 (9.0)	100 (10.1)		
**Completed posttest**				23.1 (1)	<.001
	Yes	288 (28.9)	391 (39.5)		
	No	703 (70.7)	604 (60.9)		
**Modules accessed**				342.0 (4)	<.001
	0	418 (42.2)	181 (18.2)		
	1-2	329 (33.2)	213 (21.4)		
	3-6	133 (13.4)	140 (14.1)		
	7-11	41 (4.1)	92 (9.2)		
	12	70 (7.1)	369 (37.1)		

Table 2 provides the observed means and SDs for the outcome variables across the 2 conditions, and Table 3 presents the estimates of fixed effects from the MMRM ANOVA models. There was a significant time-by-condition interaction effect for the primary outcome of PHQ-9 depression scores, indicating that participants in the intervention condition had a significantly greater reduction in depression symptoms from baseline to posttest relative to the control condition (*F*_1,701.7_=3.97; *P*=.047). For the secondary outcomes, there were also significant effects found for the intervention on symptoms of panic (Panic Disorder Screener *F_1, 706.5_*=5.59; *P*=.02) and social anxiety (Social Anxiety Disorder Screener *F*_1,680.0_=12.37; *P*<.001). Between-group effect sizes were very small for depression (*d*=0.06) and panic (*d*=0.08) symptoms and small for social anxiety symptoms (*d*=0.16). Although there were significant reductions over time for both conditions in symptoms of GAD and suicidal ideation, there were no significant differences between the 2 conditions from pretest to posttest. Likewise, the intervention did not have a significant effect on days out of role. The moderation analyses found no significant evidence that intervention effects were different on the basis of age, gender, educational attainment, or number of modules accessed, for any of the outcomes. Finally, overall satisfaction scores with the intervention at posttest were significantly higher in *FitMindKit* condition (mean 6.5, SD 2.0) than in the *HealthWatch* control condition (mean 5.3, SD 2.2; t_589.1_=7.0; *P*<.001).

**Table 2:** Observed means (SD) for outcome variables at baseline and posttest.

Outcome	FitMindKit (intervention)				HealthWatch (attention control)			
	Baseline		Posttest		Baseline		Posttest	
	n	Mean (SD)	n	Mean (SD)	n	Mean (SD)	n	Mean (SD)
Patient Health Questionnaire-9: depression	989	10.60 (4.95)	285	8.82 (5.68)	988	10.38 (4.93)	390	8.92 (5.57)
Generalized Anxiety Disorder Scale-7: anxiety	989	8.16 (4.38)	280	6.71 (4.70)	993	8.08 (4.41)	381	6.90 (4.72)
Panic Disorder Screener: panic	988	3.20 (2.97)	280	2.62 (2.93)	993	3.10 (2.81)	380	2.74 (3.04)
Social Phobia Screener: social anxiety	991	6.17 (3.80)	280	5.23 (4.10)	994	6.10 (3.75)	380	5.75 (4.26)
Suicidal Ideation Attributes Scale: suicidal ideation	917	6.47 (8.69)	267	5.88 (8.36)	911	6.44 (8.77)	357	5.65 (8.30)
Days out of role	991	2.77 (5.47)	280	2.69 (5.70)	995	2.72 (5.38)	379	2.45 (5.68)

**Table 3:** Estimates of fixed effects from mixed model repeated-measures models.

Outcome and source		*F* test (*df*)		*P* value	
**Patient Health Questionnaire-9** **: depression score**					
	Intercept		5768.89 (1,1424.1)		<.001
	Condition		0.28 (1,1424.1)		.60
	Time		78.23 (1,701.7)		<.001
	Condition×time		3.97 (1,701.7)		.047
**Generalized Anxiety Disorder Scale-7** **: generalized anxiety score**					
	Intercept		4607.66 (1,1366.3)		<.001
	Condition		0.51 (1,1366.3)		.48
	Time		66.79 (1,715.4)		<.001
	Condition×time		2.21 (1,715.4)		.14
**Panic Disorder Screener** **: panic score**					
	Intercept		1755.96 (1,1556.0)		<.001
	Condition		0.72 (1,1556.0)		.40
	Time		26.87 (1,706.5)		<.001
	Condition×time		5.59 (1,706.5)		.02
**Social Phobia Screener** **: social anxiety score**					
	Intercept		3717.83 (1,1613.4)		<.001
	Condition		3.14 (1,1613.4)		.08
	Time		40.50 (1,680.0)		<.001
	Condition×time		12.37 (1,680.0)		<.001
**Suicidal Ideation Attributes Scale** **: suicidal ideation score**					
	Intercept		844.95 (1,1501.4)		<.001
	Condition		0.02 (1,1501.4)		.88
	Time		11.80 (1,687.3)		.001
	Condition×time		0.06 (1,687.3)		.81
**Days out of role**					
	Intercept		388.09 (1,1458.1)		<.001
	Condition		0.06 (1,1458.1)		.80
	Time		2.05 (1,718.7)		.15
	Condition×time		0.42 (1,718.7)		.52

## Discussion

### Principal Findings

This study demonstrated that a self-guided transdiagnostic intervention was effective in reducing symptoms of depression, panic, and social anxiety when delivered directly to end users in a naturalistic setting. The effects of the intervention relative to the control condition were modest and did not extend to symptoms of generalized anxiety or suicidal ideation. This outcome is not surprising, as engagement with the trial and adherence to the intervention were both low, despite participants reporting greater satisfaction with the active intervention relative to the control. The absence of human support typically leads to lower levels of engagement in clinical trials [38-40], leading to attrition levels that are more commensurate with the naturalistic delivery of the intervention in the community than with an in-person clinical trial [28]. A previous review has also emphasized that attrition is typically higher for active interventions than control conditions because of factors such as higher expectations for change and greater cognitive demands [41].

Despite low adherence, this approach of delivering low-intensity self-guided interventions is highly scalable, as the resources required to deliver the intervention to the general community on the basis of demand are minimal. Assuming that 20% of adults experience elevated depression or anxiety symptoms [42], the trial reached more than 3% of the target population over 10 months, which would be challenging to achieve through other approaches such as recruitment through health services. The investment of time for users of the intervention was minimal, with participants completing a mean of 2.2 modules, which equates to approximately 6 to 12 minutes of engagement. Although few participants received a high dose of the intervention, it is clear that a proportion of users who received the intervention demonstrated significant benefits in terms of symptom reduction compared with those in the attention control condition. Although the differences between conditions on symptoms of mental health problems were modest at an individual level, when scaled to a population level, such modest effects are likely to have a significant impact. In addition, the high ecological validity of the trial suggests that the results are likely to be reflected outside of the research context, where programs are delivered in community settings, including directly to consumers through web-based marketing.

### Implications

Given the demand for the program, the evidence of benefit, and the limited resources required to deliver self-guided transdiagnostic interventions such as *FitMindKit*, there appears to be a case for making such programs publicly available, with potential for impact at a population level when delivered at scale. In particular, individuals with mild to moderate symptoms who are reluctant to engage with mental health services but recognize a need for support may benefit from self-guided transdiagnostic interventions [40]. Similarly, individuals with limited mental health literacy or high levels of mental illness stigma may find that such interventions overcome these barriers [43]. Self-guided interventions may act as a gateway to more formal psychological services, providing users with exposure to the kinds of strategies they would likely encounter when engaging with a mental health professional [40,44]. This is particularly the case for accessible programs such as *FitMindKit* that use video and brief modules focused on the core elements of CBT delivered in a flexible way determined by the user, in contrast to programs that adapt the 50-minute psychological consultation model into a web-based setting. Transdiagnostic internet interventions may fill an important gap in the delivery of evidence-based services, particularly in regions that are rural or economically disadvantaged, where traditional services may be difficult to access [45].

The differential effects of the intervention for different types of mental health symptoms may reflect the features of the intervention or the types of people who engage in internet-based programs. The strongest effects were seen for social anxiety symptoms, which may reflect the considerable treatment delays seen among people with social anxiety disorder, which typically span 16 years [5]. That is, internet-based self-help interventions may be the only evidence-based support that many people with social anxiety are willing to access. The lack of effect for GAD symptoms may suggest that additional content focused on worry in addition to rumination may be beneficial. Suicidal ideation may be more difficult to address using a transdiagnostic intervention without a specific focus on suicide and distress reducing strategies and may require a higher level of support in the context of internet interventions [35,46]. Functional impairment was also not significantly decreased by the intervention, although it is possible that the time scale for reducing functional outcomes may be lengthy, requiring longer-term follow-up.

### Strengths and Limitations

This study was one of the first to test the efficacy of a self-guided transdiagnostic web-based intervention. The strengths of the study include the large population-based sample, rigorous design, and high ecological validity of the methodology. However, the ecological validity was also a weakness because of the high rates of attrition and low adherence. Adherence was lower in the active condition than in the control condition, which may suggest that the control program may have been less cognitively demanding. Alternatively, the low adherence in the CBT intervention may reflect the challenges of engaging in video content. The analytical method used was robust to attrition, assuming data were missing at random (ie, missing on the basis of the observed characteristics of the sample). Previous research has indicated that differential attrition has little impact on the estimate of intervention effects [47], particularly when unbiased statistical methods are used that account for all available data [37]. Nevertheless, further evaluation of the intervention’s efficacy and exploration of the factors that support its uptake may be warranted. The sample was not fully representative of the population of interest, with an overrepresentation of females in particular. Although females experience a higher prevalence of mental health conditions, the underrepresentation of males may reflect ongoing challenges in engaging males in therapy, particularly in digital interventions [48]. By necessity, outcomes were assessed using brief but accurate self-report measures, which may not reflect specific clinical outcomes. The naturalistic design of the study precluded medium- or long-term follow-up, so the duration of impact could not be determined. Finally, the effect sizes were very small, suggesting that the intervention may have had minimal clinical impact for many users. Future research may benefit from investigating subgroups of the population who are most suited to brief self-guided interventions.

### Conclusions

A self-guided transdiagnostic intervention delivered through the internet without human contact was effective in reducing mental health symptoms. Although adherence was low and the effects of the intervention were modest, this study demonstrates the potential public health benefits of delivering low-intensity mental health programs at scale directly to consumers through the internet.

## Appendix

Multimedia Appendix 1 CONSORT-EHEALTH checklist (V 1.6.1).

## Abbreviations

ANOVA: analysis of variance
CBT: cognitive behavioral therapy
DQ5: Distress Questionnaire-5
DSM: Diagnostic and Statistical Manual of Mental Disorders
GAD: generalized anxiety disorder
GAD-7: Generalized Anxiety Disorder Scale-7
MMRM: mixed model repeated measures
NHMRC: National Health and Medical Research Council
PHQ-9: Patient Health Questionnaire-9
RCT: randomized controlled trial
SIDAS: Suicidal Ideation Attributes Scale
